# Critical COVID-19 and neurological dysfunction - a direct comparative analysis between SARS-CoV-2 and other infectious pathogens

**DOI:** 10.5935/0103-507X.20220229-en

**Published:** 2022

**Authors:** Ana Teixeira-Vaz, José Afonso Rocha, David Almeida e Reis, Mafalda Oliveira, Tiago Simões Moreira, Ana Isabel Silva, Matilde Monteiro-Soares, José Artur Paiva

**Affiliations:** 1 Physical Medicine and Rehabilitation Department, Centro Hospitalar Universitário de São João, Faculdade de Medicina, Universidade do Porto - Porto, Portugal.; 2 Rede de Investigação em Saúde, Centro de Investigação em Tecnologias e Serviços de Saúde, Faculdade de Medicina, Universidade do Porto - Porto, Portugal.; 3 Intensive Care Medicine Department, Centro Hospitalar Universitário de São João, Faculdade de Medicina, Universidade do Porto - Porto, Portugal.

**Keywords:** SARS-CoV-2, COVID-19, Respiratory distress syndrome, Coronavirus infections, Neurological manifestations, Pyramidal tract, Intensive care

## Abstract

**Objective:**

To evaluate whether critical SARS-CoV-2 infection is more frequently associated with signs of corticospinal tract dysfunction and other neurological signs, symptoms, and syndromes, than other infectious pathogens.

**Methods:**

This was a prospective cohort study with consecutive inclusion of patients admitted to intensive care units due to primary infectious acute respiratory distress syndrome requiring invasive mechanical ventilation > 48 hours. Eligible patients were randomly assigned to three investigators for clinical evaluation, which encompassed the examination of signs of corticospinal tract dysfunction. Clinical data, including other neurological complications and possible predictors, were independently obtained from clinical records.

**Results:**

We consecutively included 54 patients with acute respiratory distress syndrome, 27 due to SARS-CoV-2 and 27 due to other infectious pathogens. The groups were comparable in most characteristics. COVID-19 patients presented a significantly higher risk of neurological complications (RR = 1.98; 95%CI 1.23 - 3.26). Signs of corticospinal tract dysfunction tended to be more prevalent in COVID-19 patients (RR = 1.62; 95%CI 0.72 - 3.44).

**Conclusion:**

Our study is the first comparative analysis between SARS-CoV-2 and other infectious pathogens, in an intensive care unit setting, assessing neurological dysfunction. We report a significantly higher risk of neurological dysfunction among COVID-19 patients. As such, we suggest systematic screening for neurological complications in severe COVID-19 patients.

## INTRODUCTION

Coronavirus disease 2019 (COVID-19) poses a severe health threat on a global scale. Most infected patients are asymptomatic or paucisymptomatic. Nevertheless, up to 15% have severe disease, and approximately 5% become critically ill.^(1)^

This virus causes mainly respiratory signs and symptoms, whose seriousness greatly determines the severity and mortality of the disease. Nevertheless, neurological signs, symptoms and syndromes have been reported in the full clinical spectrum of COVID-19.^(1,2)^ Descriptions include olfactory and gustatory dysfunction, cranial nerve and peripheral neuropathies, signs of corticospinal tract dysfunction (CSTD), cognitive impairment, *delirium*, seizures, meningitis, encephalitis, myelitis and acute cerebrovascular disease.^(2-5)^

It remains unclear whether neurological dysfunction is solely an epiphenomenon of respiratory illness or directly related to severe acute respiratory syndrome coronavirus 2 (SARS-CoV-2) infection.^(3,5)^ The paucity of comparative studies designed to assess neurological dysfunction between COVID-19 and non-COVID-19 patients is the main reason for the persistence of this gap in knowledge.

We aimed to identify whether SARS-CoV-2 is more frequently associated with signs of CSTD and other neurological signs, symptoms, and syndromes, than other pathogens causing severe respiratory failure.

## METHODS

### Study design and definitions

This was a prospective cohort study with consecutive inclusion of patients admitted to four intensive care units (ICUs) of an intensive care department in a tertiary-care center between May 2020 and September 2021.

Inclusion criteria were age older than 18 years old and ICU admission diagnosis of infectious acute respiratory distress syndrome (ARDS), requiring invasive mechanical ventilation (IMV) for more than 48 hours.

Acute respiratory distress syndrome was defined in accordance with the Berlin definition as an acute syndrome of lung inflammation and increased alveolar-capillary permeability associated with severe hypoxia and bilateral infiltrates on chest radiographs, without evidence of left heart failure.^(6)^

Exclusion criteria were the presence of previous known central or peripheral neurologic pathologies reported in electronic clinical records (ECR) and death or discharge before the first 24 - 72 hours after ventilatory weaning.

The study was approved by our institutional review board (*Comissão de Ética* of the *Centro Hospitalar Universitário de São João* of the *Faculdade de Medicina, Universidade do Porto -* n° 169/20) and performed in accordance with the Helsinki Declaration. Written informed consent was waived considering the study setting, so verbal consent was obtained before clinical evaluation.

Sampling consisted of consecutive inclusion of all eligible patients until the calculated sample size was achieved.

A COVID-19 ARDS case was assumed when a positive result on a real-time reverse-transcriptase polymerase chain reaction (RT‒PCR) assay of nasal and pharyngeal swab specimens was found during the first 24 hours after hospital admission. Other infectious ARDS cases were assumed when there was a primary ARDS due to other pathogens (identified through respiratory tract samples, blood, or urine cultures), with a negative RT‒PCR assay for SARS-CoV-2.

Aphasia was defined as an impairment of comprehension or formulation of language, including semantic, grammar, phonology, morphology or syntax impairments.^(7)^ Dysarthria was considered when there was a motor speech impairment causing slowness, weakness and/or imprecision in speech ability^(8)^ and dysphonia when there was an impairment in voice production.^(9)^ Focal weakness was assumed when there was a muscle strength deficit involving one or more limbs.^(10)^ *Delirium* was defined as an alteration of attention, consciousness, and cognition, with a reduced ability to focus, sustain or shift attention.^(11)^ A seizure was considered a change in the level of consciousness, behavior, memory, or feelings related to uncontrolled and/or abnormal electrical activity of the brain.^(12)^ In accordance with the American Heart Association (AHA),^(13,14)^ cerebrovascular diseases were classified as: (1) transient ischemic attack (TIA), a transient episode of neurological dysfunction caused by focal brain ischemia; (2) ischemic stroke, when there was an episode of neurological dysfunction caused by focal cerebral infarction and (3) hemorrhagic stroke, when a focal accumulation of blood within the brain parenchyma or ventricular system, that was not caused by trauma, occurred. Encephalopathy refered to dysfunction of the level or contents of consciousness due to brain dysfunction, possibly resulting from global brain insults or a focal lesion in relation to primary neurological or systemic conditions.^(15)^ Encephalitis was assumed when there was an acute infection of brain parenchyma characterized clinically by fever, headache, and an altered level of consciousness,^(16)^ and myelitis when there was an inflammatory disorder of the spinal cord, characterized by acute or subacute dysfunction affecting the motor, sensory, and/or autonomic systems.^(17)^ Peripheral neuropathies encompassed disorders of peripheral nerve cells and fibers, including mononeuropathies, multifocal neuropathies and polyneuropathies.^(18)^

### Data collection methods

The main investigator, supported by two senior physicians of physical medicine and rehabilitation (PMR) and intensive care medicine, was responsible for assessing the ECR of all patients admitted to the ICU daily. This assessment was used to identify patients fulfilling eligibility criteria for the study and to evaluate the timing of their ventilatory weaning (withdrawal from ventilatory support). All patients were extubated at the time of the clinical evaluation. Data from patients who fulfilled eligibility criteria were gathered on a database, and each patient received a code number to secure their anonymity.

Eligible patients, 24 - 72 hours after ventilatory weaning, were randomly assigned through a computer-generated allocation sequence to one of three independent investigators for clinical assessment. The investigators were blinded to the patients’ characteristics and to the study research question and aims. These investigators were PMR physicians with specific training on critical care and neurological rehabilitation. To ensure common evaluation methods, an educational session taught by a board certificated PMR specialist was attended before the study began. Clinical evaluation included assessment of level of sedation (using Richmond Agitation-Sedation Scale - RASS) and the evaluation of signs of CSTD, namely enhanced deep tendon reflexes (DTR) and the Babinski sign. Each patient was evaluated by the same investigator in the first 24 - 72 hours after ventilatory weaning and re-evaluated every 24 - 72 hours, until three observations were completed.

Deep tendon reflexes were evaluated using a predefined T-shaped reflex hammer at the following locations: biceps, triceps, brachioradialis, patellar and Achilles tendons. The grading of reflex response was performed in accordance with an adapted form of the National Institute of Neurological Disorders and Stroke (NINDS) myotatic reflex scale as follows: 0 - absent, 1 - hyporeflexia, 2 - normoreflexia, 3 - hyperreflexia, 4 - hyperreflexia with unsustained clonus (< 5 beats), and 5 - hyperreflexia with sustained clonus (> 5 beats).^(19)^

The Babinski sign was evaluated using the reflex hammer dull point by running up, with light pressure, the lateral plantar side of the foot, from heel to toe. The response of each hallux and toe was recorded as extensor (Babinski sign), flexor or neutral.^(20)^

Both on DTR and on Babinski sign evaluations, when in doubt, the investigators repeated each evaluation up to three times, recording the most consistent response. The investigators registered the anonymized measurements through an anonymized electronic form.

### Outcomes and predictors

The primary outcomes were the presence of signs of CSTD and the presence of other neurological signs, symptoms, or syndromes (aphasia, dysarthria, dysphonia, focal weakness, *delirium*, seizures, stroke, transient ischemic attack, encephalopathy, encephalitis, myelitis, peripheral neuropathies). Signs of CSTD were defined as the presence of a Babinski sign in at least one extremity or hyperreflexia in at least two extremities.^(21)^ We considered that signs of CSTD were present when identified in all clinical evaluations. Information regarding other neurological signs, symptoms or syndromes was recorded from the ECR. First, we analyzed the presence of each neurological complication individually. Moreover, we performed further analysis considering a combined dichotomic endpoint (neurological dysfunction composite). This composite considered, for each patient, the presence of at least one neurological sign, symptom, or syndrome, regardless of the number of neurological manifestations.

Several other data were extracted from the ECR by the main investigator before assessing data regarding clinical examination, namely, age; sex; previous autonomy on daily-life activities assessed through the modified Rankin scale (mRS); comorbidities (hypertension, diabetes mellitus, hyperlipidemia, obesity, smoking habits, atrial fibrillation, ischemic heart disease, heart failure, peripheral vascular disease, chronic pulmonary obstructive disease, asthma, sleep apnea, psychiatric pathology, oncologic pathology and immunosuppression); number of days in the ICU and total length of stay; number of days under IMV, noninvasive ventilation and oxygen therapy; need for prone sessions; need, type and number of days under extracorporeal membrane oxygenation (ECMO); and need and number of days under renal replacement therapy, vasopressors, sedanalgesics, neuromuscular blockers and corticosteroids.

The presence of other complications during the ICU stay was also recorded. Cardiovascular complications included bradyarrhythmia, tachyarrhythmia (atrial fibrillation, flutter, other tachyarrhythmias), tachycardia-bradycardia syndrome, secondary myocardial injury, cardiac arrest, pericarditis, pericardial effusion, endocarditis, acute heart failure and cardiogenic chock. Abdominal complications included hepatitis, elevated liver enzymes, gastrointestinal bleeding, pseudo-obstruction and obstruction, diarrhea, and constipation. Infectious complications were considered when ICU-acquired infections were observed, irrespective of admission diagnosis. Muscular weakness was assessed six to nine days after ventilatory weaning through the Medical Research Council-Sum Score (MRC-SS).

### Sample size calculation and statistical analysis

Due to a lack of data on the characteristics and significance of DTR assessment in the ICU setting, data from a general population study were used.^(4,22)^ Considering an expected prevalence of the unexposed of 0.36, a relative risk (RR) of 2, a power of 80% and a level of significance of 0.05, we estimated a total sample size of 54 patients (27 per group).

Statistical analysis was performed using Statistical Pakage for the Social Sciences (SPSS) software, version 27. Categorical variables are summarized as frequencies and percentages, and continuous variables are summarized as the mean and standard deviation (variables with normal distribution) or median and interquartile range (variables with skewed distributions). Normal distribution was checked using histogram visual analysis. The chi-square test or Fisher’s exact test was used, as appropriate, to compare categorical variables. Continuous variables were compared between groups using independent samples t test or the Mann‒Whitney U test, in accordance with the variable distribution. The RR, its standard error and its 95% confidence interval (95%CI) were calculated according to Altman et al.^(23)^ Binary logistic univariate analysis was also performed, using the composite of neurological complications as the dependent variable. All reported p values are two-tailed, with a p value < 0.05 indicating statistical significance.

## RESULTS

A total of 207 patients diagnosed with primary infectious ARDS were admitted to the ICU during the study period. Were excluded 153 patients: 89 died, 42 were transferred to other hospitals before neurological assessment, and 22 had previous neurological pathology. Fifty-four patients were consecutively included in accordance with the sample size calculation: 27 with ARDS due to COVID-19 and 27 with ARDS due to other infectious pathogens.

Regarding the group with ARDS due to other infectious pathogens, most agents (56%) were *Gram*-negative bacteria (*Serratia, Rickettsia, Pseudomonas, Moraxella, Legionella, Klebsiella, Escherichia coli, Haemophilus influenzae*), but other agents, including gram-positive bacteria (*Staphylococcus, Streptococcus, Enterococcus*) and fungal pathogens (*Pneumocystis, Aspergillus*), were also identified.

Table 1 details the sample’s sociodemographic and clinical characteristics. Despite being comparable in most characteristics, COVID-19 patients were immunosuppressed less often (0% *versus* 26%: p value = 0.010). Immunosuppression in the ARDS due to other infectious pathogens group was due to posttransplant status (n = 3), alveolar proteinosis (n = 1), ANCA-MPO vasculitis (n = 1), neoplasms (n = 1) and human immunodeficiency virus infection (n = 1).

**Table 1:** Sociodemographic and clinical features

	Total (n = 54)	COVID-19 ARDS (n = 27)	Other infectious ARDS (n = 27)	p value
Sociodemographic features				
Age	62 ± 12	65 ± 12	59 ± 13	0.07^*^
Men	38 (70)	18 (67)	20 (74)	0.55†
Modified Rankin scale				1.00‡
0	53 (98)	27 (100)	26 (96)	
3	1 (2)	0 (0)	1 (4)	
Comorbidities				
Hypertension	25 (46)	14 (52)	11 (41)	0.41†
*Diabetes Mellitus*	22 (41)	11 (41)	11 (41)	1.00†
Hyperlipidemia	19 (35)	13 (48)	6 (22)	0.05†
Obesity	15 (28)	11 (41)	4 (15)	0.05†
Smoking habits	11 (20)	3 (11)	8 (30)	0.09‡
Atrial fibrillation	4 (7)	3 (11)	1 (4)	0.61‡
Ischemic heart disease	3 (6)	0 (0)	3 (11)	0.24‡
Cardiac insufficiency	5 (9)	1 (4)	4 (15)	0.35‡
Peripheral vascular disease	6 (11)	5 (19)	1 (4)	0.19‡
CPOD	6 (11)	1 (4)	5 (19)	0.19‡
Asthma	3 (6)	3 (11)	0 (0)	0.24‡
Sleep apnea	7 (13)	2 (7)	5 (19)	0.42‡
Psychiatric disorders	11 (20)	7 (26)	4 (15)	0.31†
Cancer	10 (19)	5 (19)	5 (19)	1.00†
Immunosuppression	7 (13)	0 (0)	7 (26)	0.01‡
Characteristics regarding critical respiratory illness				
APACHE	20 ± 7	19 ± 5	22 ± 8	0.14^*^
SAPS II	44 ± 16	40 ± 15	47 ± 16	0.15^*^
Days on ICU	17 (185)	22 (185)	13 (42)	0.01§
Total length of stay	36 (228)	38 (228)	31 (69)	0.09§
Days under IMV	11 (157)	14 (157)	9 (36)	0.04§
Days under NIV	2 (13)	3 (13)	1 (7)	0.06§
Days under HFNO therapy	0.5 (30)	1 (10)	0 (30)	0.11§
Prone position	31 (57)	21 (78)	10 (37)	< 0.01†
ECMO	10 (19)	6 (22)	4 (15)	0.48†
Days under ECMO	27 (146)	63 (146)	17 (31)	0.26§
Corticosteroids	33 (61)	22 (82)	11 (41)	< 0.01†
Days under corticosteroids	7 (28)	10 (24)	0 (28)	< 0.01§
Vasopressor need	48 (89)	24 (89)	24 (89)	1.00†
Days under vasopressor support	5 (75)	7 (75)	5 (22)	0.16§
Renal replacement therapy	9 (17)	3 (11)	6 (22)	0.47‡
Days under renal replacement therapy	14 (53)	23 (47)	10 (40)	0.38§
Neuromuscular block > 24 hours	49 (91)	26 (96)	23 (85)	0.35‡
Days under neuromuscular block	5 (93)	7 (93)	4 (9)	0.09§
Sedoanalgesia	54 (100)	27 (100)	27 (100)	---
Days under sedoanalgesia	11 (184)	15 (184)	9 (32)	0.03§

ARDS - acute respiratory distress syndrome; CPOD - chronic pulmonary obstructive disease; APACHE - Acute Physiology and Chronic Health Evaluation; SAPS - Simplified Acute Physiology Score; ICU - intensive care unit; IMV - invasive mechanical ventilation; NIV - noninvasive ventilation; HFNO - high flow nasal oxygen; ECMO - extracorporeal membrane oxygenation; IQR - interquartile range.

* Independent samples t test; † Pearson chi square test; ‡ Fisher exact test; § Mann-Whitney U test. Results expressed as mean ± standard deviation; n (%); or median (interquartile range).

Regarding characteristics related to critical respiratory illness (Table 1), the groups were also comparable. Nevertheless, COVID-19 patients had a significantly higher number of days in the ICU (p value = 0.006) under IMV (p value = 0.039), sedoanalgesia (p value = 0.025), and corticosteroids (p value = 0.004), with higher rates of prone positioning (p value < 0.001).

Regarding the primary outcome, 61% of the sample presented at least one neurological sign, symptom, or syndrome. Nevertheless, each neurological complication was *per se* rare (< 5%), except for *delirium* (30%) and signs of CSTD (33%).

We compared the groups on the presence of each neurological complication and on the neurological dysfunction composite. We identified significant differences between groups when analyzing the composite (p value = 0.002), with COVID-19 patients presenting with an RR 1.98-fold higher (95%CI 1.23 - 3.26) than patients admitted for ARDS due to other etiologies (85% *versus* 43%). In the analysis of each complication, our data did not reach statistical significance (Table 2). Moreover, 44% of COVID-19 patients presented signs of CSTD, while in non-COVID patients, its prevalence was 27%. Although signs of CSTD tended to be more prevalent in COVID-19 patients (RR = 1.62; 95%CI 0.72 - 3.44), this difference did not reach statistical significance (p value = 0.20). Regarding the RASS at the time of neurological examination, no differences were found between groups for any of the evaluations (p values: 1^st^ observation: 0.649; 2^nd^ observation: 0.093; 3^rd^ observation: 0.170).

**Table 2:** Complications of critical respiratory illness

	Total (n = 54)	COVID-19 ARDS (n = 27)	Other infectious ARDS (n = 27)	RR (95%CI)	p value
Neurological complications					
*Delirium*	16 (30)	9 (33)	7 (26)	1.28 (0.56 - 2.95)	0.55^*^
Seizures	2 (4)	2 (7)	0 (0)	5.00 (0.25 - 99.52)	0.49†
Transient ischemic attack	1 (2)	1 (4)	0 (0)	3.00 (0.13 - 70.54)	1.00†
Encephalopathy	1 (2)	1 (4)	0 (0)	3.00 (0.13 - 70.54)	1.00†
Encephalitis	0 (0)	0 (0)	0 (0)	--	--
Myelitis	0 (0)	0 (0)	0 (0)	--	--
Peripheral neuropathy	2 (4)	2 (7)	0 (0)	5.00 (0.25 - 99.52)	0.49†
Aphasia	1 (2)	1 (4)	0 (0)	3.00 (0.13 - 70.54)	1.00†
Dysarthria or dysphonia	1 (2)	1 (4)	0 (0)	3.00 (0.13 - 70.54)	1.00†
Focal weakness	0 (0)	0 (0)	0 (0)	--	--
Signs of CSTD‡	18 (33)	11 (44)	7 (27)	1.62 (0.72 - .44)	0.20^*^
Composite of neurological complications§	33 (61)	22 (85)	11 (43)	1.98 (1.23 - 3.26)	< 0.01^*^
Overlap with other infections	30 (56)	23 (85)	7 (26)	3.29 (1.70 - 6.34)	< 0,01^*^
Abdominal complications	22 (41)	11 (41)	11 (41)	1.00 (0.53 - 1.90)	1.00^*^
Cardiovascular complications	15 (28)	10 (37)	5 (19)	1.53 (0.68 - 3.45)	0.13^*^

ARDS - acute respiratory distress syndrome; RR - relative risk; 95%CI - 95% confidence interval; CSTD - corticospinal tract dysfunction.

* Pearson chi square test; † Fisher exact test; ‡ defined as the presence of the Babinski sign in at least one extremity or other pyramidal tract signs in at least 2 extremities(21); § including all described neurological complications. Results expressed as n (%).

To identify factors potentially associated with neurological complications, we performed a univariate analysis (Table 3), in which no other variables, apart from COVID-19 diagnosis, were associated with this adverse event.

**Table 3:** Univariable logistic analysis of possible factors associated with neurological complications

Composite for neurological complications	Odds ratio (95%CI)	p value^*^
Age	1.03 (0.99 - 1.08)	0.16
Men	2.00 (0.60 - 6.64)	0.26
Modified Rankin scale	--	--
Hypertension	0.78 (0.25 - 2.39)	0.66
Diabetes Mellitus	0.73 (0.23 -2.28)	0.59
Hyperlipidemia	2.05 (0.60 - 7.05)	0.25
Obesity	0.91 (0.27 - 3.11)	0.89
Smoking habits	0.70 (0.16 - 3.05)	0.63
Atrial fibrillation	1.97 (0.19 - 20.32)	0.57
Ischemic heart disease	0.29 (0.03 - 3.43)	0.33
Cardiac insufficiency	0.93 (0.14 - 6.12)	0.94
Peripheral vascular disease	0.13 (0.01 - 1.25)	0.08
CPOD	---	---
Asthma	1.27 (0.10 - 14.9)	0.85
Sleep apnea	0.41 (0.08 - 2.08)	0.29
Psychiatric disorders	1.17 (0.29 - 4.64)	0.83
Cancer	2.52 (0.47 - 13.6)	0.28
Immunosuppression	0.59 (0.11 - 3.24)	0.54
APACHE	1.01 (0.93 - 1.11)	0.67
SAPS II	0.98 (0.95 - 1.02)	0.44
Days on ICU	1.01 (0.99 - 1.03)	0.25
Total length of stay	0.96 (0.96 - 1.00)	0.21
Days under IMV	1.01 (0.98 - 1.03)	0.59
Days under NIV	1.20 (0.94 - 1.54)	0.15
Days under oxygen therapy	1.09 (0.91 - 1.30)	0.32
Prone position	0.97 (0.31 - 3.04)	0.96
ECMO	1.31 (0.29 - 5.95)	0.73
Days under ECMO	1.01 (0.95 - 1.05)	0.59
Corticosteroids	1.91 (0.61 - 5.97)	0.27
Days under corticosteroids	1.00 (0.92 - 1.09)	0.93
Vasopressor need	1.71 (0.31 - 9.42)	0.54
Days under vasopressor support	1.00 (0.96 - 1.04)	0.98
Renal replacement therapy	2.52 (0.47 - 13.58)	0.28
Days under renal replacement therapy	1.68 (0.65 - 4.35)	0.28
Neuromuscular block > 24 hours	2.65 (0.40 - 17.44)	0.31
Days under neuromuscular block	1.03 (0.96 - 1.10)	0.47
Sedoanalgesia > 24 hours	---	---
Days under sedoanalgesia	1.01 (0.99 - 1.03)	0.49
MRC-SS	1.03 (0.97 - 1.09)	0.31
COVID-19 ARDS	5.73 (1.65 - 19.91)	< 0.01

95% CI - 95% confidence interval; CPOD - chronic pulmonary obstructive disease; APACHE - Acute Physiology and Chronic Health Evaluation; SAPS - Simplified Acute Physiology Score; ICU - intensive care unit; IMV - invasive mechanical ventilation; NIV - noninvasive ventilation; ECMO - extracorporeal membrane oxygenation; MRC-SS - Medical Research Council Sum Score; ARDS - acute respiratory distress syndrome.

* Obtained through binary logistic analysis.

Moreover, we analyzed whether there were differences between groups in nonneurological complications (Table 2). No significant differences were observed, except for infectious complications (p value < 0.001): COVID-19 patients had a 3.29-fold higher risk (95%CI 1.70 - 6.34).

## DISCUSSION

Critical COVID-19 patients presented a 1.98-fold higher risk of developing neurological complications than patients admitted to the ICU for other infectious ARDS. To our knowledge, this is the first study comparing the presence of signs of CSTD and other neurological signs, symptoms and syndromes, between COVID-19 and non-COVID-19 critical ARDS patients.

Several signs, symptoms and syndromes of neurological dysfunction have been reported in up to 80% of COVID-19 patients on the disease’s full clinical spectrum. These findings have generated considerable concern due to their possible impact on mortality, morbidity, disability, and quality of life.^(24)^

Clinical and preclinical data have shown that SARS-CoV-2 has some degree of neurotropism, and different mechanisms have been suggested to account for this.^(25,26)^ First, it is thought that SARS-CoV-2 exploits the angiotensin converting enzyme 2 receptor to enter cells, namely, in the respiratory system and in neurological tissue.^(26)^ Nonetheless, non angiotensin converting enzyme 2 pathways have not been excluded. Both a direct transsynaptic route via the olfactory bulb and a blood circulatory pathway, through which systemic inflammation compromises the blood‒brain barrier, have been proposed.^(27)^ Another possible explanation is that the combination of hypoxia and neuroinflammation damages hippocampal and cortical areas, resulting in neuropsychiatric effects.^(25)^

Indeed, multiple studies have addressed the frequency and characteristics of neurological dysfunction among COVID-19 patients,^(28,29)^ but there is still a substantial gap in knowledge in several domains, specifically regarding critically ill patients. Both central and peripheral nervous system involvement have been extensively reported in ICU patients, either as a manifestation of systemic critical illness or its treatment.^(30)^ Furthermore, one in three patients admitted due to nonneurological pathology in the ICU develop neurological complications, which doubles the length of stay and mortality rate, increasing postdischarge disability.^(30)^ As such, critical COVID-19 patients could be prone to develop not only possible disease-associated neurological dysfunction (neuro-COVID) but also ICU-related neurological complications.

In our study, both groups were comparable regarding most baseline characteristics. Nonetheless, COVID-19 patients were immunosuppressed less often than non-COVID-19 patients. Additionally, COVID-19 patients had a higher number of days in the ICU, under IMV and under sedoanalgesia. As these factors could possibly influence the rates of neurological dysfunction, we analyzed whether any were associated with neurological dysfunction, and no significant differences were found. Additionally, when assessing the between-group differences in the RASS, we also aimed to identify the impact of those characteristics on the clinical status at the time of clinical examination, and again, no significant differences were found. Thus, COVID-19 patients had a higher risk of neurological complications regardless of the critical illness severity or its treatment.

In our sample, the overall prevalence of neurological dysfunction was 85% among COVID-19 patients, similar to the literature.^(24)^ Each neurological complication was *per se* rare, which is also similar to the Deana et al. study.^(31)^ Nevertheless, *delirium* (33%) and signs of CSTD (44%) were common complications, with a lower prevalence in comparison to other studies.^(32)^ Regarding *delirium*, despite its frequency and impact, the use of screening tools remains low, leading to a potential underestimation of its real prevalence in our sample. Additionally, regarding the signs of CSTD, its prevalence in our sample was also lower than that in previous studies, which could be due to the application of different diagnostic criteria (in relation to the absence of a standard).

In our study, we assessed signs of CSTD as an objective measure of neurological involvement. In the ICU, a detailed neurological examination can be extremely difficult to perform. However, the evaluation of signs of CSTD can be an important tool since it does not require patient collaboration, which is frequently compromised in this setting. DTR assessment allows a rapid and clear distinction between upper and lower motor neuron pathology (enhanced and depressed/absent reflexes, respectively), and the presence of the Babinski sign is a characteristic finding of upper motor neuron pathology.^(33)^ Few studies have evaluated the prevalence of CSTD in the ICU setting and its clinical relevance, as well as the real influence of iatrogenesis (namely, neuromuscular blockage) on this manifestation.^(4,34,35)^ Moreover, intensive care unit acquired weakness (ICUAW), which can have an increased prevalence in this population as risk factors are significantly more common, can also mask the presence of signs of CSTD because, when ICUAW is present, the DTR response is reduced or absent, so an underestimation of CSTD can be present in our analysis given the study setting.^(4,36-38^ However, magnetic resonance imaging studies of COVID-19 patients showed that corticospinal tract lesions were the most common lesions of the white matter.^(39)^ Indeed, in our analysis, COVID-19 patients tended to have higher rates of CSTD signs, with a 1.2-fold higher RR.

Regarding ICU complications, there were no differences in the rates of muscular weakness, cardiovascular and abdominal complications, data consistent with the literature.^(40)^

We highlight that this is the first comparative study in the ICU setting, aimed at assessing neurological dysfunction, that established a direct comparison between ARDS due to COVID-19 and other pathogens. The study design and methodological strengths reinforce our major findings. To assure the external validity of our results, there was a consecutive sampling of participants and inclusion of patients from different ICU. Regarding the internal validity of our data, we stress that the main investigator collected the predictive variables before assessing data regarding neurological bedside examination and that the three associated investigators were independent (so blinded to the patients’ characteristics). Additionally, regarding the evaluation method, the same material (to decrease the risk of instrumental biases) was used, and the investigators were trained by the same expert to ensure common evaluation techniques. Additionally, to evaluate possible confounding factors, we performed a univariate analysis that confirmed that no factor other than COVID-19 diagnosis was significantly associated with the presence of neurological dysfunction.

Our study presents some limitations. Sample size was calculated based on CSTD rates in the general population, given the absence of studies in ICU patients when our study’s recruitment started. Nevertheless, Helms et al.’s data, published during our recruitment period, reveals a higher prevalence of signs of CSTD in the COVID-19 population, which is probably related to the heterogeneity of the criteria for defining CSTD.^(4)^ Indeed, in critical ARDS patients, neuromuscular blockage is used as a standard of care, and ICUAW is present in more than 50% of cases. As the effect of both on the DTR response is thought to be its reduction or abolition, this should be fully considered when these examinations are performed in the ICU.^(34,35)^ Moreover, in our sample size calculation, we considered an RR of 2, so the absence of differences in our population regarding the CSTD signs may be because the relative risk is 19% lower than expected. As such, CSTD rates in the ICU setting may be lower, and thus, the sample size may have been underestimated. Additionally, DTR assessment and rating are dependent on subjective judgment and are operator dependent, implying inter- and intraobserver variability, the extent of which has been rarely reported.^(41)^ Moreover, the signs, symptoms and syndromes of neurological dysfunction included in the composite (aphasia, dysarthria, dysphonia, focal weakness, *delirium*, seizures, stroke, transient ischemic attack, encephalopathy, encephalitis, myelitis, peripheral neuropathies) were only considered when registered in medical records; thus, registration bias must be considered.

Given the higher percentage of neurological dysfunction among COVID-19 patients, we suggest that patients with severe forms of COVID-19 should be systematically screened for neurological complications. Moreover, it is thought that patients with neurological complications during index hospitalization have significantly worse 6-month functional outcomes.^(42)^ Early evaluation of physical medicine and rehabilitation may allow early diagnosis of neurological complications and implementation of tailored therapeutic interventions to reduce the long-term impact of these sequelae.^(32,42)^

Further studies are warranted to assess the long-term impact of neurological dysfunction in COVID-19 patients. The relationship between the signs of CSTD and neurological syndromes, as well as inter- and intraobserver reliability for CSTD, remains to be characterized in the ICU setting. Additionally, a causal relationship between disease severity and frequency and the characteristics of neurological involvement remains controversial.

## CONCLUSION

In brief, critical COVID-19 patients presented a significantly higher risk of developing neurological complications than patients admitted to the intensive care unit due to other infectious acute respiratory distress syndromes. Thus, we suggest that patients with severe forms of COVID-19 should be systematically screened for neurological complications due to its impact on patient morbidity and quality of life.

## References

[1] Guan WJ, Ni ZY, Hu Y, Liang WH, Ou CQ, He JX, Liu L, Shan H, Lei CL, Hui DSC, Du B, Li LJ, Zeng G, Yuen KY, Chen RC, Tang CL, Wang T, Chen PY, Xiang J, Li SY, Wang JL, Liang ZJ, Peng YX, Wei L, Liu Y, Hu YH, Peng P, Wang JM, Liu JY, Chen Z, Li G, Zheng ZJ, Qiu SQ, Luo J, Ye CJ, Zhu SY, Zhong NS, China Medical Treatment Expert Group for Covid-19 (2020). Clinical characteristics of coronavirus disease 2019 in
China. N Engl J Med.

[2] MadaniNeishaboori A, Moshrefiaraghi D, Mohamed Ali K, Toloui A, Yousefifard M, Hosseini M (2020). Central nervous system complications in COVID-19 patients; a
systematic review and meta-analysis based on current
evidence. Arch Acad Emerg Med.

[3] Correia AO, Feitosa PW, Moreira JL, Nogueira SA, Fonseca RB, Nobre ME (2020). Neurological manifestations of COVID-19 and other coronaviruses:
a systematic review. Neurol Psychiatry Brain Res.

[4] Helms J, Kremer S, Merdji H, Clere-Jehl R, Schenck M, Kummerlen C (2020). Neurologic features in severe SARS-CoV-2
infection. N Engl J Med.

[5] Stiller K (2013). Physiotherapy in intensive care: an updated systematic
review. Chest.

[6] Ranieri VM, Rubenfeld GD, Thompson BT, Ferguson ND, Caldwell E, Fan E, ARDS Definition Task Force (2012). Acute respiratory distress syndrome: the Berlin
Definition. JAMA.

[7] Le H, Lui MY (2022). StatPearls.

[8] Duffy JR (c2005). Motor speech disorders: substrates, differential diagnosis, and
management.

[9] Neighbors C, Song SA (2022). StatPearls.

[10] Tarulli A, Tarulli A (2016). Neurology: A Clinician’s Approach.

[11] Ramírez Echeverría MD, Schoo C, Paul M (2022). StatPearls.

[12] Huff JS, Murr N (2022). StatPearls.

[13] Sacco RL, Kasner SE, Broderick JP, Caplan LR, Connors JJ, Culebras A, Elkind MS, George MG, Hamdan AD, Higashida RT, Hoh BL, Janis LS, Kase CS, Kleindorfer DO, Lee JM, Moseley ME, Peterson ED, Turan TN, Valderrama AL, Vinters HV, American Heart Association Stroke Council, Council on Cardiovascular
Surgery and Anesthesia, Council on Cardiovascular Radiology and Intervention, Council on Cardiovascular and Stroke Nursing, Council on Epidemiology and Prevention, Council on Peripheral Vascular Disease, Council on Nutrition, Physical Activity and Metabolism (2013). An updated definition of stroke for the 21st century: a statement
for healthcare professionals from the American Heart Association/American
Stroke Association. Stroke.

[14] Easton JD, Saver JL, Albers GW, Alberts MJ, Chaturvedi S, Feldmann E, Hatsukami TS, Higashida RT, Johnston SC, Kidwell CS, Lutsep HL, Miller E, Sacco RL, American Heart Association, American Stroke Association Stroke Council, Council on Cardiovascular Surgery and Anesthesia, Council on Cardiovascular Radiology and Intervention, Council on Cardiovascular Nursing, Interdisciplinary Council on Peripheral Vascular Disease (2009). Definition and evaluation of transient ischemic attack: a
scientific statement for healthcare professionals from the American Heart
Association/American Stroke Association Stroke Council; Council on
Cardiovascular Surgery and Anesthesia; Council on Cardiovascular Radiology
and Intervention; Council on Cardiovascular Nursing; and the
Interdisciplinary Council on Peripheral Vascular Disease. The American
Academy of Neurology affirms the value of this statement as an educational
tool for neurologists. Stroke.

[15] Erkkinen MG, Berkowitz AL (2019). A clinical approach to diagnosing encephalopathy. Am J Med.

[16] Roos KL (1999). Encephalitis. Neurol Clin.

[17] Frohman EM, Wingerchuk DM (2010). Clinical practice. Transverse myelitis. N Engl J Med.

[18] Hammi C, Yeung B (2022). StatPearls.

[19] Litvan I, Mangone CA, Werden W, Bueri JA, Estol CJ, Garcea DO (1996). Reliability of the NINDS Myotatic Reflex Scale. Neurology.

[20] Isaza Jaramillo SP, Uribe Uribe CS, García Jimenez FA, Cornejo-Ochoa W, Alvarez Restrepo JF, Román GC (2014). Accuracy of the Babinski sign in the identification of pyramidal
tract dysfunction. J Neurol Sci.

[21] Álvarez N, Díez L, Avellaneda C, Serra M, Rubio MA (2018). Relevance of the pyramidal syndrome in amyotrophic lateral
sclerosis. Neurologia (Engl Ed).

[22] Lim KS, Bong YZ, Chaw YL, Ho KT, Lu KK, Lim CH (2009). Wide range of normality in deep tendon reflexes in the normal
population. Neurology Asia.

[23] Altman DG (1991). Practical statistics for medical research.

[24] Park J, Kwon YS, Kim HA, Kwon DH, Hwang J, Jang SH (2021). Clinical implications of neurological comorbidities and
complications in ICU patients with COVID-19. J Clin Med.

[25] Steardo L, Steardo L Jr, Zorec R, Verkhratsky A (2020). Neuroinfection may contribute to pathophysiology and clinical
manifestations of COVID-19. Acta Physiol (Oxf).

[26] Li YC, Bai WZ, Hirano N, Hayashida T, Taniguchi T, Sugita Y (2013). Neurotropic virus tracing suggests a membranous-coating-mediated
mechanism for transsynaptic communication. J Comp Neurol.

[27] Wu Y, Xu X, Chen Z, Duan J, Hashimoto K, Yang L (2020). Nervous system involvement after infection with COVID-19 and
other coronaviruses. Brain Behav Immun.

[28] Severo Bem Junior L, do Rego Aquino PL, Nunes Rabelo N, do Rego Aquino MA, Veiga Silva AC, Ferreira Valenca Mota RC (2020). SARS-CoV-2 and nervous system - neurological manifestations in
patients with COVID-19: a systematic review. J Neurol Res.

[29] Misra S, Kolappa K, Prasad M, Radhakrishnan D, Thakur KT, Solomon T (2021). Frequency of Neurologic manifestations in COVID-19: a systematic
review and meta-analysis. Neurology.

[30] Bleck TP, Smith MC, Pierre-Louis SJ, Jares JJ, Murray J, Hansen CA (1993). Neurologic complications of critical medical
illnesses. Crit Care Med.

[31] Deana C, Verriello L, Pauletto G, Corradi F, Forfori F, Cammarota G (2021). Insights into neurological dysfunction of critically ill COVID-19
patients. Trends Anaesth Crit Care.

[32] Helms J, Kremer S, Merdji H, Schenck M, Severac F, Clere-Jehl R (2020). Delirium and encephalopathy in severe COVID-19: a cohort analysis
of ICU patients. Crit Care.

[33] Emos MC, Rosner J (2022). StatPearls.

[34] Tezcan B, Turan S, Özgök A (2019). Current use of neuromuscular blocking agents in intensive care
units. Turk J Anaesthesiol Reanim.

[35] Murray MJ, DeBlock H, Erstad B, Gray A, Jacobi J, Jordan C (2016). Clinical Practice Guidelines for Sustained Neuromuscular Blockade
in the Adult Critically Ill Patient. Crit Care Med.

[36] Bax F, Lettieri C, Marini A, Pellitteri G, Surcinelli A, Valente M (2021). Clinical and neurophysiological characterization of muscular
weakness in severe COVID-19. Neurol Sci.

[37] Kress JP, Hall JB (2014). ICU-acquired weakness and recovery from critical
illness. N Engl J Med.

[38] Nordon-Craft A, Moss M, Quan D, Schenkman M (2012). Intensive care unit-acquired weakness: implications for physical
therapist management. Phys Ther.

[39] Parsons N, Outsikas A, Parish A, Clohesy R, D’Aprano F, Toomey F (2021). Modelling the anatomic distribution of neurologic events in
patients with COVID-19: a systematic review of MRI findings. AJNR Am J Neuroradiol.

[40] Scarpino M, Bonizzoli M, Lazzeri C, Lanzo G, Lolli F, Ciapetti M (2021). Electrodiagnostic findings in patients with non-COVID-19- and
COVID-19-related acute respiratory distress syndrome. Acta Neurol Scand.

[41] Manschot S, Van Passel L, Buskens E, Algra A, van Gijn J (1998). Mayo and NINDS scales for assessment of tendon reflexes: between
observer agreement and implications for communication. J Neurol Neurosurg Psychiatry.

[42] Frontera JA, Yang D, Lewis A, Patel P, Medicherla C, Arena V (2021). A prospective study of long-term outcomes among hospitalized
COVID-19 patients with and without neurological
complications. J Neurol Sci.

